# Directory of Public Datasets for Youth Mental Health to Enhance Research Through Data, Accessibility, and Artificial Intelligence: Scoping Review

**DOI:** 10.2196/73852

**Published:** 2025-09-08

**Authors:** Hua Min, Xia Jing, Cui Tao, Joel E Williams, Sarah F Griffin, Christianne Esposito-Smythers, Bruce Chorpita

**Affiliations:** 1 Department of Health Administration and Policy, College of Public Health, George Mason University, Fairfax, VA, United States; 2 Department of Public Health Sciences, College of Behavioral, Social and Health Sciences, Clemson University, Clemson, SC, United States; 3 Department of Artificial Intelligence and Informatics, Mayo Clinic, Jacksonville, FL, United States; 4 Department of Psychology, College of Humanities and Social Sciences, George Mason University, Fairfax, VA, United States; 5 Department of Psychology, University of California, Los Angeles, 1285 Franz Hall, Box 951563, Los Angeles, CA, 90095, United States, 1 3107941262

**Keywords:** youth mental health, publicly available datasets, data accessibility, data integration, artificial intelligence, evidence-based research

## Abstract

**Background:**

Youth mental health issues have been recognized as a pressing crisis in the United States in recent years. Effective, evidence-based mental health research and interventions require access to integrated datasets that consolidate diverse and fragmented data sources. However, researchers face challenges due to the lack of centralized, publicly available datasets, limiting the potential for comprehensive analysis and data-driven decision-making.

**Objective:**

This paper introduces a curated directory of publicly available datasets focused on youth mental health (less than 18 years old). The directory is designed to serve as critical infrastructure to enhance research, inform policymaking, and support the application of artificial intelligence and machine learning in youth mental health research.

**Methods:**

Unlike a systematic review, this paper offers a brief overview of open data resources, addressing the challenges of fragmented health data in youth mental health research. We conducted a structured search using 3 approaches: targeted searches on reputable health organization websites (eg, National Institutes of Health [NIH] and Centers for Disease Control and Prevention [CDC]), librarian consultation to identify hard-to-find datasets, and expert knowledge from prior research. Identified datasets were curated with key details, including name, description, components, format, access information, and study type, with a focus on freely available resources.

**Results:**

A curated list of publicly available datasets on youth mental health and school policies was compiled. While not exhaustive, it highlights key resources relevant to youth mental health research. Our findings identify major national survey series conducted by organizations such as the NIH, CDC, Substance Abuse and Mental Health Services Administration (SAMHSA), and the U.S. Census Bureau, which focus on youth mental health and substance use. In addition, we include data on state and school health policies, offering varying scopes and granularities. Valuable health data repositories such as ICPSR, Data.gov, Healthdata.gov, Data.CDC.gov, OpenFDA, and Data.CMS.gov host a wide range of research data, including surveys, longitudinal studies, and individual research projects.

**Conclusions:**

Publicly accessible health data are essential for improving youth mental health outcomes. Compiling and centralizing these resources streamlines access, enhances research impact, and informs interventions and policies. By improving data integration and accessibility, it encourages interdisciplinary collaboration and supports evidence-based interventions.

## Introduction

Many mental health conditions develop during youth and adolescence, making it a global public concern [1-3]. The number of young people experiencing mental health issues continues to rise. According to the Centers for Disease Control and Prevention (CDC), the percentage of high school students reporting feelings of sadness and hopelessness increased significantly over the past decade, rising from 28% in 2011 to 40% in 2023 [4]. This marked increase highlights a growing mental health crisis among adolescents, emphasizing the urgent need for prevention and early intervention. Adolescence is a period of significant psychological, emotional, and social development, making it a critical window for addressing mental health concerns [5]. When left unaddressed, these issues can lead to long-term worse consequences, including impaired educational attainment, strained relationships, and adverse health outcomes in adulthood, far beyond adolescence. In 2021, the U.S. Office of the Surgeon General released an advisory titled “Protecting Youth Mental Health,” emphasizing the urgent need to address the mental health challenges faced by young people [6].

Young individuals with substance use disorders are at an elevated risk of developing co-occurring mental health challenges, including suicidal behaviors, which can exacerbate lifelong difficulties, contribute to social issues, and result in poorer treatment outcomes [7,8]. Preventable mental health issues, such as adolescent suicide [9,10] and substance use [11,12], significantly contribute to youth disease, disability, and mortality, highlighting the importance of proactive measures in urgent need to save lives and improve the well-being of young people. Suicide, one of the leading causes of death among young people, is often preventable through the timely identification of warning signs, access to supportive networks, and evidence-based interventions [10]. Similarly, substance use disorders, which frequently emerge during adolescence, can be reduced through early preventive strategies that focus on education, community support, and behavioral health services [12]. Investing in preventive mental health care during adolescence yields far-reaching benefits [13], not only for affected individuals at the time, but also for them later in life, their families, communities, and societies. Effective prevention programs can enhance resilience, reduce stigma, and promote mental well-being, ultimately fostering healthier and more productive generations overall.

A significant gap persists between the mental health needs of children and adolescents in the United States and the availability of services to meet those needs [14]. Most young people with mental health conditions do not seek or receive treatment [15]. Barriers include limited awareness of available services, inaccessibility, insurance coverage issues, insufficient coordinated care, a shortage of specialized providers, unstable living conditions, concerns about confidentiality, and fear of stigmatization [15,16]. Schools have been identified as critical settings for screening, preventing, and treating youth mental health issues, as students spend the majority of their daily time in these environments [5,17]. To address this critical issue, it is essential to strengthen youth-focused mental health services, integrate mental health education into school curricula, and equip educators, parents, and peers with tools to recognize and respond to early signs of distress [5]. By prioritizing prevention and early treatment, we can disrupt the cycle of untreated mental health issues and cultivate supportive environments where young people can thrive.

In alignment with these efforts, evidence-based medicine continues to drive advancements in mental health care [18]. Evidence-based approaches are crucial for addressing youth mental health challenges [19]. To ensure high-quality, impactful research and promote its translation into clinical practice, service delivery, and policy, the National Institute of Mental Health (NIMH) developed its strategic plan for research aiming at achieving these goals [20]. This plan addresses emerging challenges and opportunities by leveraging scientific advances to improve mental health outcomes. Among its 4 goals, Goal 2—“Examine Mental Illness Trajectories Across the Lifespan”—closely aligns with the collection and analysis of mental health data using diverse datasets.

Mental health conditions are multifaceted [21], influenced by a combination of biological factors (eg, brain trauma and genetics), psychological factors (eg, stress and traumatic events), and environmental, social, and economic determinants. Understanding these complexities demands the collection and integration of diverse datasets to capture the full spectrum of influences on mental health, especially for youth. By assembling data that capture biological, psychological, social, and environmental factors, researchers can reveal a more comprehensive picture of mental health conditions, discover patterns, identify at-risk populations, and design targeted interventions that address the unique needs of young people. Notable existing data sources include national surveys such as the Youth Risk Behavior Surveillance System (YRBSS) [22], the National Survey on Drug Use and Health (NSDUH) [23], and the National Survey of Children’s Health (NSCH) [24], large-scale longitudinal studies such as the Adolescent Brain and Cognitive Development (ABCD) study [25], and targeted clinical trials [26] exploring the efficacy of early interventions and prevention strategies.

The growing volume of biomedical data generated by these studies provides a solid foundation for data mining and knowledge discovery to extract meaningful insights across diverse data types [27-30]. The new emerging artificial intelligence (AI) techniques [28], such as machine learning (ML), natural language processing, and especially generative AI [29], are increasingly used to analyze large-scale datasets, identify early indicators of mental health issues, and develop predictive models for conditions such as depression, anxiety, suicide, and substance use disorders. For example, AI is being used to process real-time data from wearable devices [31], social media activities [32], and electronic health records [28] to gain insights into behavioral patterns and risk factors among adolescents. In the mental health domain, AI has been increasingly applied to develop high-quality predictive models and perform advanced data analyses. For example, deep learning techniques have been used with structural magnetic resonance imaging data from large-scale studies such as the ABCD to predict biological sex and identify gender-related variations in brain structure [33]. In addition, AI-driven analyses have been used to assess the effectiveness of telehealth interventions using datasets such as the NSDUH and other Substance Abuse and Mental Health Services Administration (SAMHSA) resources [34].

One significant challenge in data science and AI is the lack of accessible health care data. Electronic health record data are often restricted due to privacy and security regulations, such as Health Insurance Portability and Accountability Act (HIPAA). This limitation highlights the critical role publicly available datasets play in enabling big data analysis and advancing research. These datasets are particularly vital in advancing youth mental health research, where access to comprehensive data is crucial for revealing meaningful insights and discovering new interventions.

With the National Institutes of Health (NIH) data sharing policy, an increasing amount of research data is now available for secondary data reuse, enabling a broader range of studies to build upon existing findings. Datasets such as the YRBSS, NSDUH, NSCH, and ABCD study provide researchers with open access to comprehensive data on behavioral, social, and biological factors that influence mental health. Effectively integrating these datasets will be crucial in deepening our understanding of mental health and developing innovative solutions for prevention and treatment. By leveraging these resources, researchers can collaborate across institutions, validate findings, and accelerate the discovery of new insights. Furthermore, publicly available data facilitate the development of AI-powered tools to identify trends, predict risks, and personalize mental health interventions. This approach can lead to scalable, equitable solutions to address the growing mental health needs of young people worldwide.

This paper aims to review existing publicly available datasets related to youth mental health and provide a detailed directory as a reference tool for researchers interested in integrating diverse datasets for advanced data analysis or AI-assisted discovery across different aspects of youth mental health. By highlighting the scope and potential of these datasets, this paper seeks to guide future research efforts in leveraging diverse data sources to better understand and address the complex challenges facing youth mental health.

## Methods

**Figure 1.**
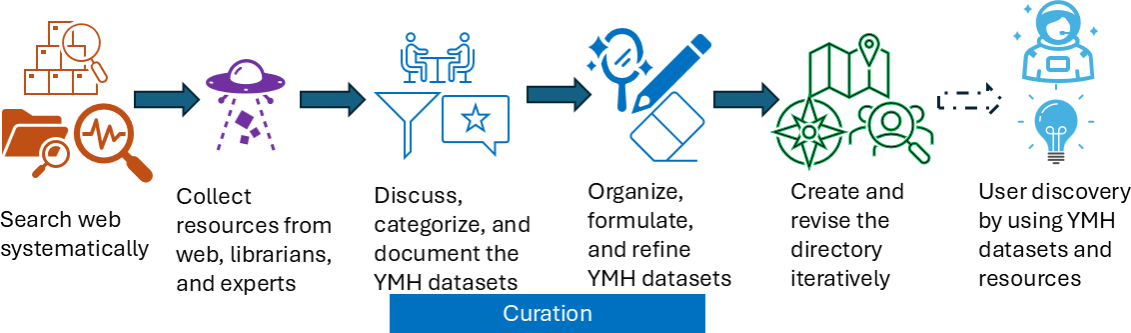
Overall workflow of curating the youth mental health data directory (dashed arrow indicates anticipated usage of the directory). YMH: youth mental health.

In this brief review, we used a multifaceted approach to identify publicly available datasets relevant to youth mental health. As the study does not involve human participants or identifiable personal information, ethics approval was not required, consistent with the guidance from the U.S. Department of Health and Human Services Office for Human Research Protections (45 CFR 46). Figure 1 presents the overall workflow, which includes conducting a structured web search, consulting with librarians, and incorporating knowledge shared by domain experts. The search results were then curated through organizing, formulating, and refining the identified datasets. The resulting directory has the potential to support discovery and evidence-based research in youth mental health.

The structured search included 3 main approaches: (1) targeted search: we conducted focused searches of reputable websites of health organizations, institutions, and government agencies (eg, NIH and CDC). Our search focused primarily on U.S.-based resources; (2) librarian consultation: we collaborated with professional librarians specializing in health and social sciences to identify and verify datasets not readily found in publicly available search engines; and (3) expert knowledge: leveraging our team’s expertise and prior familiarity with youth mental health research, we included datasets we had previously worked with or identified as significant in the field.

For our targeted search, we began with a literature review on PubMed using Medical Subject Headings (MeSH) terms. We conducted 2 separate searches: the first focused on mental health and the second on substance use.

- Mental health search: (“Datasets as Topic”[MeSH]) AND (“Mental Health”[MeSH]) OR (“Behavioral Medicine”[MeSH])
  Filters: Child (birth-18 years), Child (6‐12 years), Adolescent (13‐18 years)
- Substance use search: (“Behavioral Medicine”[Majr]) OR (“Mental Health”[Majr]) AND (“Substance-Related Disorders”[Majr])
  Filters: Child (birth-18 years), Child (6‐12 years), Adolescent (13‐18 years)

The inclusion criteria were as follows: (1) the article was peer reviewed and written in English; (2) the full text was available; (3) the research was conducted using publicly available datasets, including studies using multiple datasets; and (4) the primary focus was on mental health or substance use. We also included datasets focused on school health policies and systems that, while not containing individual-level mental health data, provide essential contextual information relevant to youth mental health research. These datasets support investigations of structural and environmental influences on mental health outcomes and were included to encourage more integrative and upstream approaches in the field.

The search returned 197 papers for mental health and 294 papers for substance use. All papers were manually reviewed by the first author (HM), who extracted the datasets mentioned in the articles. The results were validated by the second author (XJ). After identifying the datasets, we visited the corresponding websites directly to obtain more detailed information about each dataset. We also consulted our university librarians and one of the authors (CES), an expert in youth mental health, to identify and add additional relevant datasets.

Next, we curated and categorized the identified datasets, providing a detailed and consistent description of each, including the following elements:

1. Dataset name: the title of the dataset and the organizations responsible for its creation.
2. Description: the primary research goals and questions the dataset is designed to address.
3. Components: key variables and types of data collected, such as biological, psychological, social, or economic data.
4. Downloadable site: direct URL link to download the dataset, with an emphasis on datasets that are freely available and do not require restrictive licensing or complex application processes.
5. Data format: information about the format of the data (eg, CSV, SAS, SPSS, and so on).
6. Data categories: the type of study or data collection method, such as national surveys, administrative records, or longitudinal cohort studies.
7. Access requirements: public-use data are openly accessible without restrictions. Restricted-use data typically require a Data Use Agreement (DUA), an approved proposal, and access through a secure data center or administrative process. These requirements are often necessary to access sensitive variables such as detailed geographic information or personally identifiable data.

## Results

A list of publicly available datasets addressing youth mental health and school policies was compiled in Table 1. While this is not a comprehensive list of all available datasets, it includes key resources that are relevant to youth mental health. The CDC initiates, conducts, and supports many national surveys to understand youth mental health, including the YRBSS, School Health Profiles (SHP), School Health Policies and Practices Study (SHPPS), National Health Interview Survey (NHIS), and the National Youth Tobacco Survey (NYTS). Another important agency is the SAMHSA [35] which collects data related to substance use and mental health services, including NSDUH, National Substance Use and Mental Health Services Survey (N-SUMHSS), Mental Health Client-Level Data (MH-CLD), and Treatment Episode Data Set (TEDS). The U.S. Census Bureau also conducts an annual household survey, the NSCH, which provides national- and state-level data on the health and health care needs of children aged 0‐17 years, as well as their families and communities. The NIMH Data Archive is a valuable resource for mental health data, including studies focusing on youth mental health. One such study is the ABCD study, which tracks over 11,000 children starting at ages 9‐10 to examine how factors such as substance use and genetics influence brain development. Another is the Treatment for Adolescents with Depression Study (TADS), along with its follow-up, Substance Use and Other Outcomes Following Treatment for Adolescent Depression (SOFTAD), evaluates treatments for adolescent depression and their long-term effects on substance use and other outcomes. In addition, the Agency for Healthcare Research and Quality (AHRQ) conducts the annual Medical Expenditure Panel Survey (MEPS) to collect data on health care use, costs, insurance coverage, and health status from individuals, families, medical providers, and employers across the United States. The National Data Archive on Child Abuse and Neglect (NDACAN) curates a wide range of datasets related to child maltreatment, including administrative records, survey results, and state- and local-level reports. These datasets provide valuable insights into the prevalence, characteristics, and outcomes of child abuse and neglect, as well as the effectiveness of related systems and services.

**Table 1.** Overview of publicly available datasets addressing youth mental health and school policies.

Name	Administered by	Description	Components	Reference	File format	Category	Access requirements
Youth Risk Behavior Surveillance System (YRBSS)	CDC^a^ Division of Adolescent and School Health (DASH)	Since 1991, the YRBSS has been administered during the spring of odd-numbered years to students in grades 9‐12 enrolled in U.S. public and private schools.	Focuses on youth health behaviors and conditions, including sexual activity, injury and violence, bullying, diet, physical activity, obesity, mental health, suicide-related behaviors, and substance use.	[36]	Access and ASCII	Cross-sectional and nationally representative survey	Public-use data
School Health Profiles (SHP)	CDC DASH	Since 1996, the survey has been conducted every even-numbered year to assess school health policies and practices in states, large urban school districts, and U.S. territories.	Covers topics such as health education, physical education and activity, tobacco use prevention, nutrition services, school health policies, family and community involvement, school-based health services, and professional development.	[37]	ASCII, SAS, or Excel	Cross-sectional survey	Restricted-use data
School Health Policies and Practices Study (SHPPS)	CDC	Since 1994, the survey has been conducted every 6 years at the state, district, school, and classroom levels for grades K–12.	Covers 8 components of school health at the state and district levels: health education, physical education and activity, health services, mental health and social services, nutrition services, safe school environment, staff health promotion, and family and community involvement.	[38]	ASCII, Access, SAS, SPSS	Cross-sectional and nationally representative survey	Public-use data
National Health Interview Survey (NHIS)	CDC through the National Center for Health Statistics (NCHS)	Since 1957, the survey has been conducted annually on the civilian noninstitutionalized population, selecting one adult (Sample Adult) and one child (Sample Child) randomly from each family.	Includes annual core topics such as chronic conditions, functioning and disability, insurance, health care access and use, health behaviors, and demographics; rotating core topics include health care use, mental health assessments, chronic pain, preventive services, industry and occupation, and injuries.	[39]	ASCII, CSV, SAS, SPSS, STATA	Cross-sectional and nationally representative survey	Public-use data and restricted-use data
National Youth Tobacco Survey (NYTS)	CDC	Since 1999, the NYTS has been conducted annually as a voluntary, school-based, self-administered survey targeting U.S. middle and high school students.	Measures tobacco-related items, including demographics, youth access, and exposure to secondhand smoke.	[40]	SAS, Access, and Excel	Cross-sectional and nationally representative survey	Public-use data
National Survey of Children’s Health (NSCH)	Health Resources and Services Administration’s (HRSA) Maternal and Child Health Bureau (MCHB) and U.S. Census Bureau	Conducted annually as a household survey, it is the largest national- and state-level survey on the health and health care needs of children aged 0‐17 years, their families, and their communities.	Covers family health and activities; health conditions and functional difficulties; insurance status, type, and adequacy; health care use and access; impact of child’s health on the family; medical home; parental health and neighborhood perceptions; physical and mental health; preventive and specialty care; school readiness; and transition to adult care.	[41]	SAS, STATA	Cross-sectional and nationally representative survey	Public-use data
National Survey on Drug Use and Health (NSDUH)	Substance Abuse and Mental Health Services Administration (SAMHSA)	Since 1971, the survey has been conducted annually and provides representative data of persons aged 12 years and older in the civilian noninstitutionalized population of the United States	Includes topics on the use of tobacco, alcohol, and drugs; substance use disorders; mental health issues; and receipt of substance use and mental health treatment	[42]	SAS, SPSS, Stata, Delimited, R	Cross-sectional and nationally representative survey	Public-use data and restricted-use data
National Substance Use and Mental Health Services Survey (N-SUMHSS)	SAMHSA	Annual survey of all active substance use and mental health facilities across the United States	Provides number, location, and characteristics of public and private-owned mental health and substance use facilities in the United States	[43]	SAS, SPSS, Stata, Delimited, R	Cross-sectional and nationally representative survey	Public-use data and restricted-use data
Mental Health Client-Level Data (MH-CLD)	SAMHSA	Since 2011, MH-CLD captures patients treated in facilities that are either operated by or receive block grant funding through a state mental health agency.	Collects demographic and mental health characteristics data on approximately 5 to 6 million patients receiving care in outpatient, hospital inpatient, and residential services each year	[44]	SAS, SPSS, Stata, Delimited, R	Administrative records data	Public-use data and restricted-use data
Treatment Episode Data Set (TEDS)	SAMHSA	TEDS is a national data system of annual admissions to and discharges from substance use treatment facilities that are licensed or certified by Single State Agencies to provide substance use treatment services.	Contains demographic, substance use, mental health, clinical, legal, and socioeconomic characteristics of all admissions and discharges aged 12 years and older who are receiving publicly funded substance use treatment services.	[45]	SAS, SPSS, Stata, Delimited, R	Administrative records data	Public-use data and restricted-use data
Adolescent Brain Cognitive Development Study (ABCD)	NIMH^b^ Data Archive	It is a prospective cohort study that enrolled 11,876 children aged 9 to 11 years, recruited from 21 study sites in the baseline year (2016‐2018). This study will follow participants until they are approximately 19‐20 years old.	Collects data on brain development, physical health, behavioral patterns, and mental health, including neuroimaging, substance use, cognitive assessments, and psychosocial factors. It also gathers genetic and epigenetic data to explore how environmental and genetic influences affect adolescent development over time.	[46]		Longitudinal cohort study	Restricted-use data
Treatment for Adolescents with Depression Study (TADS) and Substance Use and Other Outcomes Following Treatment for Adolescent Depression (SOFTAD)	NIMH Data Archive	TADS included 439 participants ages 12 to 17 years from various geographic regions in the United States who were diagnosed with major depression. The SOFTAD study recruited 196 of these 439 adolescents and followed them for an additional 3.5 years to explore whether successful treatment of depression reduces the risk of developing substance use disorders and other outcomes.	Examines the short- and long-term effectiveness of the antidepressant medication fluoxetine (Prozac), cognitive behavioral therapy alone, and their combination for treating depression in adolescents.	[47]	SAS, SPSS, CSV	Randomized longitudinal clinical trial	Restricted-use data
Medical Expenditure Panel Survey (MEPS)	Agency for Healthcare Research and Quality (AHRQ)	Started in 1996, it is an annual survey of families, individuals, medical providers (doctors, hospitals, pharmacies, etc), and employers across the United States. It includes 2 main components: Household Component and Insurance Component.	Collects data on the specific health services that Americans use, how frequently they use them, the cost of these services, and how they are paid for, as well as data on the cost, scope, and breadth of health insurance held by and available to U.S. workers.	[48]	SAS, Stata, R, ASCII	Cross-sectional and nationally representative survey	Public-use data and restricted-use data
National Data Archive on Child Abuse and Neglect (NDACAN)	U.S. Department of Health and Human Services	Since 1988, NDACAN promotes scholarly exchange among researchers in the child maltreatment field. The archive provides access to national-, state-, and local-level data.	Provides access to national-, state-, and local-level data primarily aimed at tracking the volume and nature of child maltreatment reports each year in the United States, and supports research to inform policies and practices that improve child welfare and protect children.	[49]	SAS, SPSS, and STATA	A data repository containing cross-sectional, nationally representative, and Administrative/clinical data	Restricted-use data
NKI Rockland Sample (NKI-RS)	NIH^c^ and the New York State Office of Mental Health	Approximately 1500 individuals aged 6 to 85 years were recruited from the Rockland County community in New York.	Provides a rich neuroimaging and phenotypic resource to characterize lifespan normative brain-behavior relationships. Includes psychiatric diagnostics, medical, behavioral, and cognitive phenotyping; multimodal brain imaging (resting fMRI^d^, diffusion MRI^e^, morphometric MRI, arterial spin labeling); genetics; and actigraphy.	[50] [51]	BIDS and CSV	Longitudinal study	Public-use data and restricted-use data
Healthy Brain Network (HBN)	Child Mind Institute	HBN creates a Biobank from a community sample of 10,000 children and adolescents (ages 5‐21 years) residing in the New York City area	Collects a wide array of data, including neuroimaging (MRI), genetics, behavioral assessments, and clinical data to improve understanding of brain health and mental illness across age groups.	[52] [53]	BIDS and CSV	Cross-sectional study with biobanking and some longitudinal follow-up.	Restricted-use data

a CDC: Centers for Disease Control and Prevention.

b NIMH: National Institute of Mental Health.

c NIH: National Institutes of Health.

d fMRI: functional magnetic resonance imaging.

e MRI: magnetic resonance imaging.

Like the ABCD study, there are 2 other local neuroimaging studies: the NKI Rockland Sample (NKI-RS) [54] and the Healthy Brain Network (HBN) [55]. The NKI-RS focuses on understanding brain function and structure across a wide range of conditions, while HBN aims to improve the diagnosis and treatment of mental health and learning disorders by identifying biological markers, such as brainwave signals and imaging data. Both studies provide valuable neuroimaging data to advance research in mental health and cognitive development. Image data from both studies are stored on Amazon Web Services (AWS) S3, providing accessible and scalable storage for large datasets.

The Inter-university Consortium for Political and Social Research (ICPSR) [56], based at the University of Michigan, is one of the world’s largest archives of social science data. It offers extensive datasets for research and education across social, behavioral, and health sciences. These datasets include both publicly available and restricted-access data from single studies, U.S. national surveys, and international studies and surveys. A search for “youth mental health” or “youth substance use” or “adolescent mental health” or “adolescent substance use” on ICPSR returns 362 studies. Table 2 shows 10 randomly selected public-use youth mental health datasets from the ICPSR. For example, Monitoring the Future: A Continuing Study of American Youth, funded by National Institute on Drug Abuse, is a study of the behaviors, attitudes, and values of Americans from adolescence through adulthood. To date, 133 data files are hosted at ICPSR.

**Figure 2.**
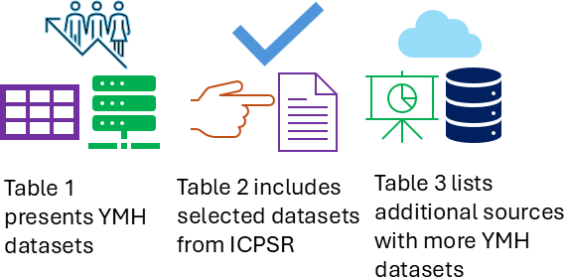
The summary view of the youth mental health data directory. ICPSR: Inter-university Consortium for Political and Social Research; YMH: youth mental health.

Other central repositories for open data from the U.S. government include Data.gov, which are presented in Table 3. The Department of Health and Human Services (HHS) publishes datasets as part of its Open Data program. The primary resource for public access to health-related data is Healthdata.gov, with additional specialized sites available for specific topics, including Data.CDC.gov, OpenFDA, and Data.CMS.gov. Figure 2 summarizes the results.

**Table 2.** Ten randomly selected public-use youth mental health datasets from Inter-university Consortium for Political and Social Research.

Name	Description	Components	Downloadable site	Data format	Category
National Longitudinal Study of Adolescent to Adult Health (Add Health), 1994‐2018	Adolescents in grades 7 through 12 were enrolled during the 1994‐1995 school year and were followed into young adulthood through 4 in-home interviews. The most recent interview was conducted in 2008, when participants were between 24 and 32 years old.	Collects data on social, economic, psychological, and physical well-being, alongside contextual information about family, neighborhood, community, school, friendships, peer groups, and romantic relationships.	[57]	SAS, SPSS, STATA, R, ASCII, Delimited	Longitudinal cohort study
Family cumulative risk and mental health in Chinese adolescents	This study examines the developmental cascades among family cumulative risk, life satisfaction, and symptoms of anxiety and depression in Chinese adolescents.	Covers family cumulative risk and mental health	[58]	SPSS	Observational study
Resilience and Mental Health among Juveniles	The first study involved 201 juveniles, the second involved 253 juveniles.	Study 1 analyzes the relationship between resilience and overall mental health of juveniles admitted to youth education centers. Study 2 examines how resilience directly and indirectly affects juveniles’ mental health.	[59]	SPSS	Observational study
Adolescent Depression	This dataset focuses on self-compassion, self-efficacy, and trait resilience as mediators between insecure attachment (specifically attachment anxiety and attachment avoidance) and depression.	Collects anxiety and depression	[60]	SPSS	Observational study
Health Behavior in School-Aged Children (HBSC), 2009‐2010	Since 1982, the WHO^a^ Regional Office for Europe has sponsored the HBSC study, surveying the health behaviors of young people every 4 years in over 40 countries. The data available here are from the results of the U.S. survey conducted during the 2009‐2010 school year.	Includes questions on substance use (tobacco, alcohol, marijuana), family composition, physical health, health behaviors (eating habits, dieting, physical activity, body image), health issues, and bullying. Also collects school administrator data on programs, policies, and health course content.	[61]	SAS, SPSS, STATA, R, ASCII, Delimited	Cross-sectional and nationally representative survey
National Youth Survey (NYS) Series	NYS is a longitudinal study of delinquency among American youth during the period 1976 through 1980. For this series, parents and youth were interviewed about events and behavior of the preceding year to gain a better understanding of both conventional and deviant types of behavior by youths.	Includes data on demographics, socioeconomic status, family disruptions, neighborhood issues, parental expectations, labeling, family and peer influences, attitudes toward deviance, parental discipline, community engagement, substance use, victimization, pregnancy, depression, outpatient services, domestic violence, and sexual activity.	[62]	SAS, SPSS, STATA, ASCII, Delimited	Longitudinal cohort study
National Survey of Children: Wave I, 1976, Wave II, 1981, and Wave III, 1987 (ICPSR 8670)	The survey aims to assess the physical, social, and psychological well-being of American children, create a national profile of their living conditions, analyze the relationship between these conditions and child development, and examine the effects of marital disruption on children and family	Provides information on the child’s well-being, family, family disruption experiences, behavior, physical health, and mental health.	[63]	ASCII	Longitudinal cohort study
Child Abuse, Neglect, and Violent Criminal Behavior in a Midwest Metropolitan Area of the United States, 1967‐1988 (ICPSR 9480)	The study examines the link between childhood abuse or neglect and later criminal behavior, focusing on whether early victimization leads to criminal offending in adolescence or adulthood, and its association with juvenile and adult arrests, particularly for violent offenses.	Part 1: Demographics (age, race, sex, birthdate). Part 2: Abuse/neglect details (type, duration, removal, placement, survival). Part 3: Family information (household, disruptions, reporters) and perpetrator data (relation, age, race, sex). Part 4: Adult arrest charges (occasion, counts, year, location, offense). Part 5: Juvenile arrest charges (year, number of arrests, offense). Parts 1‐3 focus on individuals under age 11; Part 4 covers adult charges; Part 5 covers juvenile charges.	[64]	SAS, SPSS, STATA, ASCII	Prospective cohort study
Monitoring the Future: A Continuing Study of American Youth (12th-Grade Survey), 2020 (ICPSR 38156)	This survey of 12th-grade students examines changes in values, behaviors, and lifestyle orientations of American youth. Students complete one of 6 randomly assigned questionnaires, each with unique topical questions but all including core questions on demographics and drug use.	Covers use of substances such as tobacco, alcohol, marijuana, prescription drugs, LSD, cocaine, ecstasy, heroin, and more. Also includes attitudes toward religion, women’s roles, educational goals, self-esteem, drug education, and exposure to violence and crime.	[65]	SAS, SPSS, STATA, R, ASCII, Delimited	Cross-sectional and nationally representative survey with a longitudinal subsample
Youth, Education, and Society Supplement: School Health Policies and Practices Survey (YES), 2006‐2014 (ICPSR 36350)	YES surveyed secondary schools in the Monitoring the Future study and a larger supplementary sample, conducting annual surveys of school administrators from 2006‐2007 to 2013‐2014 school year.	Includes school characteristics, nutrition and physical education policies, lunch programs, and vending machines, stores, and snack bars.	[66]	SAS, SPSS, STATA, ASCII	Cross-sectional and nationally representative survey

a WHO: World Health Organization.

**Table 3.** Important data sources from reputable organizations that include youth mental health data.

Name	Reference	Note	No. of datasets
ICPSR	[56]	Provides access to a wide range of social science datasets	More than 350,000
Data.gov	[67]	A central repository for all U.S. government data	306,135
Healthdata.gov	[68]	The U.S. Department of Health and Human Services’ platform for accessing health-related datasets	3239
Data.CDC.gov	[69]	The CDC’s^a^ open data platform	1077
OpenFDA	[70]	Provides access to FDA-related^b^ datasets	152
Data.CMS.gov	[71]	Offers datasets from the Centers for Medicare and Medicaid Services	160

a CDC: Centers for Disease Control and Prevention.

b FDA: Food and Drug Administrarion.

In addition to the raw data collected, researchers often identify, code, and summarize publicly available research reports and publications within a particular domain (eg, children and adolescent psychotherapy), categorizing them as researcher assertions rather than data assertions [72]. Such literature data, similar to systematic literature reviews, represent another critical form of publicly accessible information. Despite their descriptive nature, they can be used for meta-analyses, understanding the current state of the field, identifying potential research gaps, and conducting other aggregate evidence-based analyses. Common and reliable sources for identifying data or studies include PubMed, Cochrane Library, PsycINFO, Embase, ClinicalTrials.gov, and NIH RePORT for research portfolios.

## Discussion

### Summary of Results

Our findings highlight several major national survey series, conducted by organizations such as the CDC, SAMHSA, and the U.S. Census Bureau, that focus on youth and adolescent mental health and substance use. For instance, the YRBSS monitors behaviors that may negatively impact the health of high school students. The NSCH provides extensive data on various aspects of children’s lives, including physical and mental health, health care access and quality, and their family, neighborhood, school, and social environments. Similarly, the NSDUH offers estimates of substance use and mental illness at the national, state, and local levels, helping to identify prevalence across different groups, track trends over time, and assess treatment needs.

We also collect data on state and school health policies, which provide data with different scopes and granularities. Since youth spend the majority of their daily time at school, these policies play a crucial role in their mental health. The CDC’s Division of Adolescent and School Health (DASH) has developed an action guide to promote mental health and well-being in schools [73]. It also collects data on youth and school health policies and practices through initiatives such as the SHP. Similarly, the CDC has conducted the SHPPS to provide additional insights. These 2 datasets, SHP and SHPPS, are primarily focused on school policy and practice, rather than on individual student data or a sample of student data. However, they are important for researchers who want to study the impact of policy on student mental health, which is why we have also included them in our directory.

The World Health Organization (WHO) conducts a similar survey, the Global School-based Student Health Survey (GSHS), to help countries measure and assess behavioral risk and protective factors among young people aged 13 to 17, related to the leading causes of morbidity and mortality among children and adults worldwide.

The ABCD study, NKI-RS, and HBN are 3 influential neuroimaging studies that are contributing valuable insights into brain development and mental health. A key aspect of their success is the use of advanced storage solutions such as AWS S3, which enables them to handle large and complex datasets efficiently. By using AWS S3, these studies improve data management and provide easier access to large-scale neuroimaging data, advancing mental health research and supporting personalized, data-driven treatment approaches.

ICPSR, Data.gov, Healthdata.gov, Data.CDC.gov, OpenFDA, and Data.CMS.gov are valuable health data repositories that host a wide range of research data, including surveys, longitudinal studies, and individual research projects. These platforms provide extensive datasets that are crucial for public health research, policy analysis, and decision-making. They offer access to data on various health topics, such as disease prevalence, health care use, medication usage, and health behaviors, allowing researchers, policymakers, and practitioners to make informed decisions and conduct comprehensive analyses to improve public health outcomes.

### Evaluation of the Strengths and Limitations of Datasets in This Directory

To support researchers in selecting the most appropriate data sources, we summarize key strengths and limitations of publicly available datasets included in this directory. These evaluations highlight the robustness of mental health measures, longitudinal design, and coverage of specific subpopulations. Several datasets, such as NHIS, NYTS, NSCH, YRBSS, and NSDUH, are cross-sectional in design. While they provide nationally representative snapshots of mental health indicators, behaviors, and service use, their cross-sectional nature limits the ability to examine developmental trajectories or long-term outcomes. The YRBSS, in particular, focuses on high school students and includes mental health-relevant items such as depressive symptoms, suicidal ideation, and risk behaviors, but it does not provide clinical diagnosis or longitudinal follow-up. In contrast, datasets such as the ABCD study and MEPS offer longitudinal data, allowing researchers to track changes in mental health, cognitive development, and service use over time. The ABCD study is especially notable for its multimodal design, incorporating neuroimaging, genetics, and detailed behavioral and psychosocial assessments, making it a rich resource for studying the developmental pathways of mental health and substance use. Some datasets, for example, MH-CLD and TEDS, provide detailed clinical information on service encounters but may lack comprehensive symptom-level or diagnostic data, particularly for youth. In addition, access to certain datasets, such as those housed in the NDACAN or clinical trials such as TADS and SOFTAD, may require additional steps for approval and involve samples that are not broadly generalizable.

### Importance of Publicly Available Data for Youth Mental Health Research

Adolescence is a pivotal stage of development where mental health challenges, such as anxiety, depression, and trauma-related disorders, often emerge. However, these challenges remain under-researched due to limited access to comprehensive, high-quality datasets. Furthermore, considering youths are at the early stage of one’s life, effective management and treatment of mental health conditions will have profound impacts on the rest of their lives, which distinguishes the crucial period and critical nature of studying youth mental health.

Access to publicly available data enables researchers to identify trends and patterns in mental health conditions, risk factors, and service use among diverse populations of youth. Such data also allow for the evaluation of the impact of school health policies on mental health outcomes, shedding light on how these policies can support students’ well-being and promote mental health education. By uncovering key insights into the prevalence and effects of mental health challenges, these data guide future research and inform intervention strategies. Furthermore, they provide a foundation for developing evidence-based interventions tailored to the unique needs and characteristics of different youth groups, promoting more effective and targeted outcomes.

Publicly available data also help to address health disparities by providing a means to explore differences in mental health outcomes based on factors such as socioeconomic status, race, and geographic location. This, in turn, can inform more equitable solutions and ensure that mental health services reach those who need them most. Furthermore, open access to data fosters cross-disciplinary collaboration, bringing together experts from various fields such as psychology, public health, and education to tackle complex mental health issues from multiple angles.

Moreover, publicly available datasets empower community organizations, schools, families, and mental health providers by providing actionable insights that help shape programs, allocate resources more effectively, and create supportive environments for youth.

### Significance of Curating a List of Publicly Available Data and Resources for Youth Mental Health Research

With the growing number of studies, datasets, and reports being published across various platforms—each designed to address specific aspects of health and often collecting data independently without integration—the volume of fragmented data is increasing. This highlights the urgent need to create a centralized list of health data repositories to streamline access, reduce redundancy, and enhance their overall utility, thereby accelerating advancements in youth mental health research.

Compiling a list of available data also encourages data reuse, allowing researchers to explore secondary research questions and make the most of existing datasets. While there are similar efforts, such as ICPSR, its scope is broader and not specifically focused on youth mental health. In contrast, our directory focuses exclusively on youth mental health, helping users save time by eliminating the need to search for datasets individually. Moreover, ICPSR does not provide a comprehensive list of youth mental health datasets, omitting key resources such as the NYTS and MH-CLD.

In addition, a centralized health data repository helps policymakers by providing easy access to relevant data, empowering them to make informed, data-driven decisions to improve youth mental health outcomes. Furthermore, such a resource supports interdisciplinary collaboration by enabling researchers from diverse fields—such as public health, psychology, pediatrics, education, and social work—to access and use datasets relevant to their work. This collaborative potential can accelerate innovation, inform policy, and drive the development of targeted interventions to address complex health challenges such as youth mental health effectively.

### Benefits of Linked Datasets

The list of publicly available data and resources not only promotes secondary data analysis but also provides a foundation for more advanced linked data analysis. By integrating datasets from different sources, such as school health policies and youth mental health outcomes, researchers can gain deeper insights into the relationships between various factors influencing youth well-being. For example, Foti et al [74] investigated how state and local agencies use the YRBSS and SHP in various ways to monitor and address issues related to adolescent and school health. Gould et al [75] used 2 national surveys to assess the impact of the BP Deepwater Horizon oil rig explosion on mental health, substance use disorders, chronic health conditions, and use among local residents. The first survey was the NSDUH Gulf Coast Oversample (GCO), part of the 2011 NSDUH, and the second was the CDC’s Gulf States Population Survey (GSPS). Linked data allowed researchers to conduct more comprehensive analyses that can inform more effective interventions and policies, both locally and globally.

However, several challenges exist in linking youth mental health datasets. First, the number of well-known and widely available datasets in this field is limited. This paper aims to introduce existing datasets by compiling them from multiple sources into a centralized directory. Second, the lack of standardized frameworks hinders the integration of youth mental health data. Data collected and archived by various organizations and studies result in inconsistencies in terminology, variations in data structures, and differences in how key concepts—such as mental health outcomes, demographic information, and treatment interventions—are represented across datasets. One widely recognized solution to this challenge is the use of ontologies, which have been successfully applied in the medical field. Ontologies provide a structured framework to harmonize these core concepts, facilitating more efficient data sharing, comparison, and integration [76]. While some ontologies exist in the behavioral and mental health domain, such as the Behavior Change Intervention Ontology (BCIO) [76], Addiction Ontology [77], Mental Disease Ontology [78], Mental Health Management Ontology [79], and Social Determinants of Health Ontology (SDoHO) [80], they remain insufficient and lack detailed coverage specific to youth mental health. The absence of comprehensive standardization creates significant barriers to effective data integration and analysis, further underscoring the urgent need for ontologies in the behavioral sciences [81].

### Limitations

While the availability of publicly accessible health data is invaluable for advancing research on youth mental health, there are several limitations that must be addressed. First, our list is not comprehensive, but it serves as a starting point for researchers to discover and access relevant health data repositories. Second, there are inconsistencies across datasets. Datasets across different platforms may use varying definitions, classifications, and measurement tools for mental health conditions, making it difficult to compare or combine data from multiple sources. This lack of standardization can introduce biases and complicate analyses. Third, many publicly available datasets are cross-sectional, capturing a snapshot of youth mental health at a particular point in time. However, understanding mental health requires a longitudinal approach, tracking changes over time. The lack of long-term data restricts the ability to assess causal relationships or predict future mental health outcomes. Finally, some datasets may not adequately represent certain geographic regions or demographic groups, particularly marginalized communities. This lack of representation can hinder the understanding of mental health disparities and limit the applicability of research findings to diverse populations.

### Future Work

Despite these limitations, several avenues for future work can improve the utility and impact of publicly available data for youth mental health research. This paper represents an initial step in creating a dedicated directory for publicly available youth mental health data resources. In the future, we aim to develop a Youth Mental Health Ontology (YMHO) based on the datasets in this directory, harmonizing the data with established standards (eg, Observational Medical Outcomes Partnership [OMOP] Common Data Model [CDM] [82]) and YMHO itself. By compiling the high-quality datasets, based on defined criteria, to advance the understanding of youth mental health.

There is a need for standardized definitions, measures, and data collection methods across datasets to facilitate data sharing and integration. Such standardization would allow researchers to more effectively compare and combine data from different sources, leading to more robust findings.

Continued advances in ML and AI are expected to drive breakthroughs across a wide range of research fields, including mental health, in the coming years. However, these advancements depend on large volumes of well-curated, FAIR (Findable, Accessible, Interoperable, and Reusable) data. Effective and automatic curation processes, as shown in the central part of Figure 1, are crucial for data integration and harmonization, encompassing the characterization, annotation, management, and preservation of digital datasets. Here are several existing automatic curation examples. PubTator [83] is an AI-empowered annotation tool, a key component of automatic curation; some efforts focus on automatic population of ontology (Ontorat [84]) or streaming some parts of the workflow (ROBOT [85]) as well as other efforts [86,87]; other efforts focus on automatically identifying candidate key phrases to facilitate ontology construction and maintenance [88-91]. In addition, automatic curation could include information extraction, key component recognition, data integration, and so on. These processes will further improve the efficiency and accessibility of these valuable resources and also meet the needs of a large number of curated data for ML and AI to apply.

Building collaborative platforms that integrate multiple data sources and encourage interdisciplinary collaboration will promote innovative solutions to youth mental health issues. These platforms could also facilitate real-time data sharing, allowing for quicker responses to emerging mental health trends and crises.

### Conclusions

The availability of publicly accessible health data is a cornerstone for improving youth mental health outcomes. Compiling an extensive list of such data and resources not only streamlines access but also enhances the impact of research, interventions, and policies for youth mental health. This initiative is vital for fostering collaboration, addressing disparities, and advancing the collective goal of healthier, more resilient adolescents.

## Abbreviations

ABCD: Adolescent Brain and Cognitive Development
AHRQ: Agency for Healthcare Research and Quality
AI: artificial intelligence
AWS: Amazon Web Services
BCIO: Behavior Change Intervention Ontology
CDC: Centers for Disease Control and Prevention
CDM: Common Data Model
DASH: Division of Adolescent and School Health
DUA: Data Use Agreement
GCO: Gulf Coast Oversample
GSHS: Global School-based Student Health Survey
GSPS: Gulf States Population Survey
HBN: Healthy Brain Network
HHS: Department of Health and Human Services
HIPAA: Health Insurance Portability and Accountability Act
ICPSR: Inter-university Consortium for Political and Social Research
MEPS: Medical Expenditure Panel Survey
MH-CLD: Mental Health Client-Level Data
ML: machine learning
N-SUMHSS: National Substance Use and Mental Health Services Survey
NDACAN: National Data Archive on Child Abuse and Neglect
NHIS: National Health Interview Survey
NIH: National Institutes of Health
NIMH: National Institute of Mental Health
NKI-RS: NKI Rockland Sample
NSCH: National Survey of Children’s Health
NSDUH: National Survey on Drug Use and Health
NYTS: National Youth Tobacco Survey
OMOP: Observational Medical Outcomes Partnership
SAMHSA: Substance Abuse and Mental Health Services Administration
SDoHO: Social Determinants of Health Ontology
SHP: School Health Profiles
SHPPS: School Health Policies and Practices Study
SOFTAD: Substance Use and Other Outcomes Following Treatment for Adolescent Depression
TADS: Treatment for Adolescents with Depression Study
TEDS: Treatment Episode Data Set
WHO: World Health Organization
YMHO: Youth Mental Health Ontology
YRBSS: Youth Risk Behavior Surveillance System
